# Nutritional Contingency Reduces Alcohol Drinking by Altering Central Neurotransmitter Receptor Gene Expression in Rats

**DOI:** 10.3390/nu11112731

**Published:** 2019-11-11

**Authors:** Starr Villavasso, Cemilia Shaw, Elena Skripnikova, Krishna Shah, Jon F. Davis, Sunil Sirohi

**Affiliations:** 1 Laboratory of Endocrine and Neuropsychiatric Disorders, Division of Basic Pharmaceutical Sciences, College of Pharmacy, Xavier University of Louisiana, New Orleans, LA 70125, USA; svillava@xula.edu (S.V.); cshaw6@xula.edu (C.S.); eskripni@xula.edu (E.S.); kshah@xula.edu (K.S.); 2 Department of Integrative Physiology and Neuroscience, Washington State University, Pullman, WA 99164, USA; jon.davis@wsu.edu

**Keywords:** high-fat diet, alcohol, nutritional contingency, palatable diet, CNS gene expression

## Abstract

We have previously shown that 6 weeks of intermittent high-fat diet (Int-HFD) pre-exposure significantly reduced alcohol drinking in rats, providing preliminary evidence of the effectiveness of a dietary intervention in reducing alcohol intake. However, the functional framework and underlying neurobiological mechanisms of such dietary intervention are unknown. Here, we examined the impact of Int-HFD pre-exposure duration on alcohol drinking, plasma feeding peptides, and central neurotransmitter receptors gene expression. Male Long Evans rats (*n* = 6–7/group) received no pre-exposure, 1 or 2 weeks pre-exposure to Int-HFD and alcohol drinking (two-bottle choice) was evaluated. We observed HFD pre-exposure-dependent decrease in alcohol drinking, with a significant decrease observed following 2 weeks of Int-HFD pre-exposure. No significant between-group differences in plasma feeding peptides (i.e., ghrelin, leptin, insulin) were detected. A PCR array revealed that the expression of several neurotransmitter receptors was significantly (*p* < 0.05 and ≥2-fold) altered in the striatum and ventral tegmental area compared to controls. These data suggest that pre-exposure to a palatable diet is critical to reduce alcohol drinking in rats, possibly through genetic alterations in the brain reward circuitry. Importantly, the present study is a step forward in identifying the critical framework needed to evaluate the therapeutic potential of nutritional contingency in the management of alcoholism.

## 1. Introduction

Alcohol use disorder (AUD) is a debilitating disease afflicting ~15.1 million Americans [1] and ~3 million deaths worldwide are attributed to alcohol yearly [2]. Current FDA-approved drugs for AUD reduce alcohol consumption by producing an aversive reaction upon alcohol consumption (i.e., Disulfiram), modulating glutamatergic neurotransmission (i.e., Acamprosate), or reducing rewarding effects of alcohol by blocking opioidergic neurotransmission (i.e., Naltrexone). However, impaired emotional, physiological and metabolic status observed in patients diagnosed with AUD adversely affects treatment outcomes and ~2/3 of the patients relapse, which remains a major hurdle to the successful treatment of alcoholism [3,4,5].

Alcohol is unique among abused drugs in that it possesses calories and problematic alcohol consumption reduces nutrient intake and absorption [6]. Importantly, alcoholics have lower body weight and reduced body mass index compared to healthy controls, which signify a poor nutritional status [7,8], a condition that could contribute to AUD pathology [7,9]. Interestingly, alcoholics display an increased intake of highly palatable food during recovery [10,11]. However, it is unclear if this is an attempt to restore caloric deficits or to alleviate the negative consequences of alcohol withdrawal. In this regard, it is now clear that neuroendocrine peptides regulate both energy balance and reinforcing properties of alcohol by interacting with brain reward circuitry (reviewed in Vadnie et al., 2014). Therefore, a compromised nutritional status may further exacerbate various behavioral impairments observed in alcoholics and could substantially contribute to this chronic relapsing disorder. Improving nutritional status during abstinence may not only compensate for general malnutrition in AUD but also could serve to ameliorate some of the adverse symptoms observed in alcohol withdrawal, thereby enhancing the prospects of other behavioral and pharmacological strategies for the management of alcoholism. However, the correction of nutritional deficiencies and a balanced diet approach are often overlooked as important treatment components for AUD. Furthermore, functional components of such a dietary intervention are also unclear.

It is also important to note that the manner by which exposure to palatable diets can influence alcohol drinking behavior is poorly understood as multiple factors (e.g., macronutrient composition, exposure duration) can modulate the impact of palatable food-intake on alcohol drinking [12,13]. Therefore, it is critically important to assess the functional framework before testing such a nutritional intervention in the management of alcoholism. In this regard, a recent study from our lab indicated that six-weeks of intermittent pre-exposure to a nutritionally complete high-fat diet (HFD) attenuated alcohol drinking in rats [14,15], which has important clinical implications in the management of AUD. However, critical components (e.g., minimum effective exposure, duration of treatment, administration frequency) and underlying neurobiological mechanisms of intermittent HFD-induced reduced alcohol drinking are unknown. Therefore, the present study examined the impact of intermittent HFD pre-exposure duration on alcohol drinking, plasma feeding peptides, and central neurotransmitter receptors gene expression.

## 2. Material and Methods

### 2.1. Animals

Adult male Long-Evans rats (~250 g) were obtained from Envigo RMS, Inc (Indianapolis, IN). Rats were individually housed under controlled humidity (60%–70%) and temperature (65–70 °F) on a reverse light/dark-light cycle. Food and water were available ad libitum to all rats throughout the experiment. Animals were gently handled for one week and baseline data, including body weight, food and water intake were measured before any experimental manipulation. All animal experiments were approved by the Institutional Animal Care and Use Committee at the Xavier University of Louisiana.

### 2.2. Diets

All the rats had ad libitum access to normal rodent standard chow (Tekland-Envigo Diets #2020X, energy density-3.1 kcal/gm, fat (16%Kcal), protein (24%Kcal) and carbohydrates (60%Kcal)). In addition to chow, the experimental group received intermittent access to HFD (Research Diets #D03082706, energy density-4.54 kcal/gm, fat (40%Kcal), protein (15%kcal), and carbohydrates (46%Kcal; 8% Kcal from sucrose)). The detailed dietary composition has been presented previously [16].

### 2.3. General Procedure

**Figure 1:**
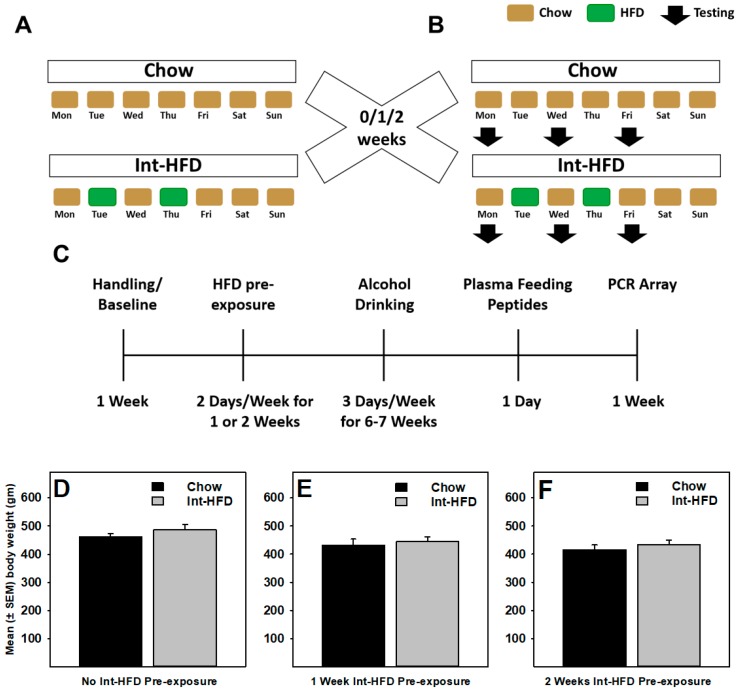
Schematics of the intermittent access to a nutritionally complete high-fat diet paradigm and body weight following testing. (**A**) Rats received intermittent exposure (24 h every Tuesday and Thursday) of a nutritionally complete high-fat diet (HFD) for 0, 1 or 2 weeks and (**B**) tested for alcohol drinking (20% *v*/*v*) on non-HFD days (Monday, Wednesday and Friday) and 24-h consumption was recorded. The intermittent HFD cycling was maintained during behavioral testing, as described in the timeline (**C**) No between-group body weight difference existed at the end of alcohol testing sessions in animals receiving (**D**) no pre-exposure (*n* = 7/group) or (**E**) 1-week (*n* = 6/group) or (**F**) 2 weeks (*n* = 6/group) pre-exposure to intermittent HFD cycling.

A total of 38 male Long Evans rats were used in the present study. Rats with no significant baseline differences in body weight, food or water intake received chow (control) or intermittent access (24 h, Tuesdays and Thursdays; Int-HFD) to a high-fat diet (see Figure 1A). A total of 6 groups (3 Int-HFD and 3 respective chow controls; *n* = 6–7/group) of rats were used. Since one of the main objectives of the study was to examine the minimum effective HFD exposure duration to reduce alcohol drinking, separate groups of rats received either no pre-exposure (0Wk-Int-HFD), one-week pre-exposure (1Wk-Int-HFD) or 2 weeks pre-exposure (2Wk-Int-HFD) of an intermittent HFD cycling before alcohol testing began. To account for different pre-exposure conditions in the experimental groups, each of these groups had their separate control groups which received chow, instead of HFD on Tuesdays and Thursdays, (0Wk-Int-Chow), (1Wk-Int-Chow) or (2Wk-Int-Chow), respectively. Normal rodent chow and water were always available ad libitum to all groups of rats. Following no pre-exposure or Int-HFD pre-exposure (1 or 2 weeks), rats were given 24-h access to unsweetened alcohol (20% *v*/*v*) on Mondays, Wednesdays, and Fridays (non-HFD days). Intermittent HFD cycling continued as described above during all testing sessions unless noted otherwise (see Figure 1B). Intermittent HFD cycling was then suspended and alcohol drinking was measured for 10 additional days. Following the completion of alcohol drinking studies, Int-HFD cycling resumed for ~9–12 days before further assessments occurred. Next, blood samples were collected by tail bleeding for feeding peptide analysis. Finally, all the rats were sacrificed and the amygdala, hypothalamus, striatum and ventral tegmental area (VTA) brains regions were dissected from snap-frozen brains for a PCR array analysis. All behavioral testing, plasma and brain collection occurred 3–6 h post-Int-HFD exposure on Chow access days.

### 2.4. Ethanol Testing

Alcohol testing was carried out as described previously [15]. Briefly, alcohol drinking behavior was assessed by providing rats with unsweetened alcohol (20% *v*/*v*) and water using a two-bottle choice paradigm for 24 h on Monday, Wednesday and Friday. Int-HFD feeding paradigm continued during ethanol testing. Water and alcohol bottle positions were alternated between alcohol testing sessions to account for conditioning effects on alcohol intake. Twenty-four-hour alcohol and water consumption were measured, along with body weight and food intake.

### 2.5. Feeding Peptide Analysis

MILLIPLEX Rat Metabolic Hormone Magnetic Bead Panel—Metabolism Multiplex Assay (Millipore Sigma # RMHMAG-84K, EMD Millipore Corporation, Massachusetts, USA) was used to simultaneously analyze Amylin (Active), C-Peptide 2, Ghrelin (Active), GIP (Total), GLP-1 (Active), Glucagon, IL-6, Insulin, Leptin, MCP-1, PP, PYY, TNF-α. Blood samples were collected in tubes containing EDTA (5ul; 0.5M), dipeptidyl peptidase (DPP-4) inhibitor (15 ul; 10mM), and Fisher Halt Protease Inhibitor Cocktail (5ul). Following blood collection, samples were centrifuged at 1000× *g* for 20 min. Plasma was transferred into a fresh tube on ice and stored at −20 ℃ until the day of further analysis. On the day of analysis, plasma samples were thawed on ice and assayed in triplicates according to the kit manufacturer’s instructions.

### 2.6. Central Neurotransmitter Receptors Gene Expression

After all the behavioral experiments were completed, rats were maintained on intermittent HFD cycling until they were euthanized, which occurred 3–6 h following the end of the Int-HFD exposure cycle. These testing only occurred in the group of rats receiving two-weeks pre-exposure to intermittent HFD cycling. Brains were isolated and snap-frozen and stored at −80 °C. On the day of analysis, hypothalamus, amygdala, striatum, and ventral tegmental area (VTA) were micro-dissected and neurotransmitters receptors gene expression was evaluated using RT^2^ Profiler PCR array. On the day of analysis, the specific brain region was placed in RNAlater (Ambion, Foster City, CA, USA). A tissue Ruptor (QIAGEN, Germantown, MD, USA), a QIAshredder (QIAGEN cat# 79654) and an RNeasy Plus mini kit (QIAGEN cat#74134) were used for total RNA extraction and isolation as per the manufacturer’s protocol. The concentration and purity of the RNA samples were determined with a Nanodrop spectrophotometer. The Purity of RNA samples (>1.9) was confirmed by the 260/280 absorbance ratio. To rule out any possibility of PCR inhibitors contamination, the PCR array has built-in positive PCR controls (PPC) monitor and reverse transcription efficiency was calculated during online data analysis by ratios between the PPC and reverse transcription control (RTC). Furthermore, the degradation and integrity were assessed by Experion Automated Electrophoresis (BioRad, Hercules, CA, USA) and all RNA samples were of high quality and passed all necessary requirements. An RT^2^ First Strand kit (QIAGEN cat# 330401) was used to synthesize cDNA from RNA (350 ng) for each sample following the manufacturer’s protocol. PCR amplification was conducted using MyiQ Real-Time quantitative PCR system (Bio-Rad). The baseline threshold was manually set to 100 RFU in primary data analysis for all arrays. The Rat RT^2^ Profiler PCR arrays (QIAGEN cat# PARN-060Z) were used to profile the expression of a total of 84 genes (Table 1). All array passed quality control tests (PCR array reproducibility, RT efficiency and genomic DNA contamination). A web-based data analysis tool (QIAGEN) was used to calculate fold change and p-values. Reference/Housekeeping genes with the least between-group variability were chosen from built-in reference genes in the PCR array. At least three reference genes (*Hprt1, Ldha, Actb, B2m and Rplp1*) per brain region were used for qPCR data normalization. The C_T_ cut-off was set to 35 and measurements >35 were excluded from further analysis. Fold Change (2^ (- Delta Delta C_T_)) is the normalized gene expression (2^(- Delta C_T_)) in the Int-HFD samples divided by the normalized gene expression (2^ (- Delta C_T_)) in the Chow samples. Fold regulation represents fold change results in a biologically meaningful way. Fold change values greater than one indicate a positive- or an up-regulation, and the fold regulation is equal to the fold change. Fold change values less than one indicate a negative or down-regulation, and the fold regulation is the negative inverse of the fold change.

### 2.7. Statistical Analysis

Food and fluid consumption were analyzed by a mixed-model two-way ANOVA with appropriate post-hoc (Bonferroni’s) analysis. The within-subject variable was time intervals (24 h measurements) and the between-group variable was Int-HFD pre-exposure (0, 1 or 2 weeks). A *t*-test compared body weight and feeding peptides data. PCR array data were analyzed, as described previously [17], using unpaired t-test as per the manufacturer-recommended Web-based RT^2^ Profiler PCR Array data analysis software and others using this method to evaluate gene expression. All statistical comparisons were conducted at 0.05 α level in GraphPad Prism 7.05.

## 3. Results

### 3.1. Intermittent HFD Exposure and Body Weight

Rats received either no pre-exposure or 1–2 weeks pre-exposure of intermittent HFD and tested for alcohol drinking while intermittent HFD cycling continued. No statistically significant (*p* > 0.05) between-group differences were observed in the body weight at the end of alcohol testing in either groups (Figure 1D–F). Similarly, no significant (*p* > 0.05) between-group body weight differences existed following either HFD pre-exposure or at the end of the study.

### 3.2. Intermittent HFD Exposure and Feeding

**Figure 2:**
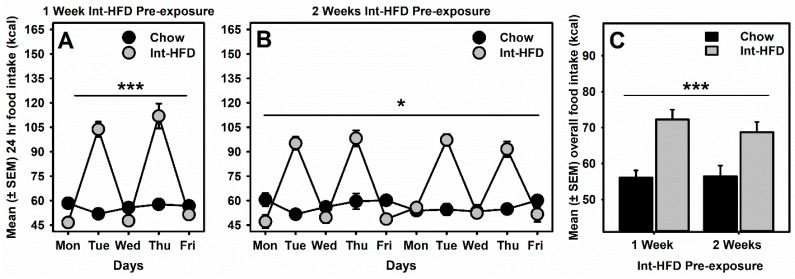
**The intermittent high-fat diet (HFD) feeding paradigm and daily energy consumption.** Mean (±SEM) of daily total food intake (kcal) was compared between the chow control group and the Int-HFD group during (**A**) 1-week and (**B**) 2-weeks intermittent HFD pre-exposure. Rats with intermittent HFD access significantly overconsumed calories on HFD exposure days. Rats with chow-only access maintained baseline energy consumption throughout the week. (**C**) Overall, energy intake was significantly elevated in Int-HFD rats on HFD access days, compared to the chow group. * *p* < 0.05, *** *p* < 0.001 main effect of intermittent HFD exposure.

A mixed-model ANOVA was used to analyze caloric intake data which identified a main effect of diet (F_1,10_ = 23.13, *p* < 0.001), time (F_4,40_ = 63.84, *p* < 0.0001), and a significant diet × time interaction (F_4,40_ = 72.24, *p* < 0.0001) in the case of animals receiving one week pre-exposure to the intermittent HFD (Figure 2A). Similarly, a main effect of diet (F_1,10_ = 9.038, *p* < 0.05), time (F_9,90_ = 47.22, *p* < 0.0001) and a significant diet×time interaction (F_9,90_ = 58.42, *p* < 0.0001) was observed in the case of animals receiving two-week pre-exposure to the intermittent HFD (Figure 2B). A post hoc analysis further identified that caloric intake was significantly (*p* < 0.0001) elevated on Tuesdays and Thursdays, whereas it was not significantly (*p* > 0.05) different on Monday, Wednesday or Friday in either one or two weeks pre-exposed groups compared to the chow controls. We also analyzed overall food intake in both groups and a mixed-model ANOVA identified only a main effect of diet (F_1,10_ = 36.52, *p* < 0.001) but no time or interaction effects. These data suggest that overall, rats receiving either one- or two-weeks pre-exposure of intermittent access to HFD significantly increased their caloric intake on the HFD access days (Tuesday and Thursday) and no significant differences in chow groups and Int-HFD groups were evident between the two exposure conditions (Figure 2C).

### 3.3. Intermittent HFD Exposure and Alcohol Drinking

**Figure 3:**
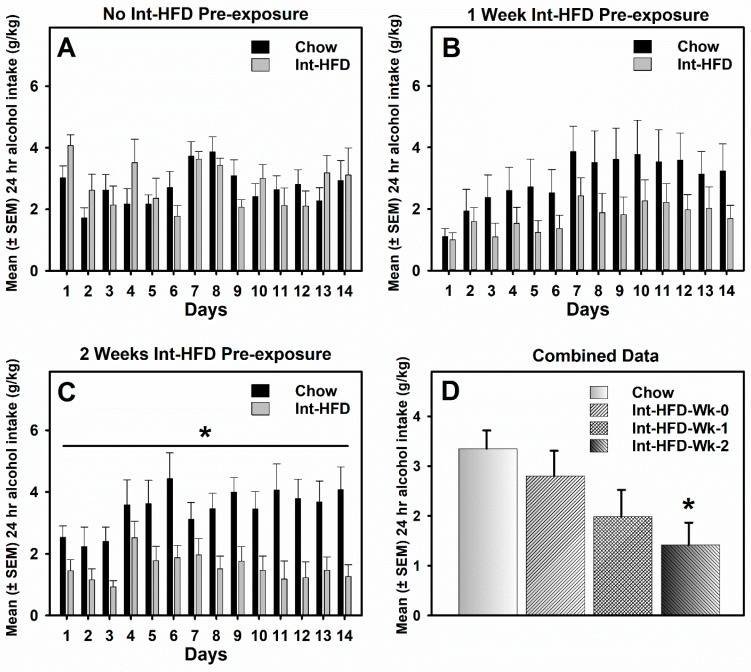
**The intermittent HFD feeding paradigm and alcohol intake.** Data compare mean (±SEM) 24 h alcohol consumption (20% *v*/*v*) for both the chow control and the Int-HFD group. Intermittent cycling on HFD was continued during the testing in all groups. (**A**) No significant between-group differences in alcohol drinking existed in the absence of intermittent HFD pre-exposure. (**B**) After 1-week of pre-exposure to intermittent HFD, a non-significant decrease in alcohol drinking was observed in the Int-HFD group compared to the chow controls. (**C**) Alcohol drinking was significantly attenuated in the Int-HFD group compared to the chow controls receiving 2 weeks of intermittent HFD pre-exposure. * *p* < 0.05, main effect of intermittent HFD exposure. (**D**) Mean (± SEM) overall alcohol consumption during the last 7 days of testing under combined controls condition. * *p* < 0.05, compared to the chow controls.

Alcohol drinking data were analyzed by a mixed-model two-way ANOVA. Alcohol drinking was not significantly different between groups during the entire testing session in the animals who did not receive intermittent HFD pre-exposure (Figure 3A) or received one-week pre-exposure (Figure 3B) to the intermittent HFD cycling. However, alcohol drinking was significantly (F_1,10_ = 7.686, *p* < 0.05) attenuated in the Int-HFD group compared to the chow controls following two-weeks pre-exposure to intermittent HFD cycling (Figure 3C). Overall, there was HFD pre-exposure dependent decrease in the alcohol drinking with a ~40% decrease observed in the case of one-week and a ~55% decrease observed following two-weeks intermittent HFD cycling as analyzed by one-way ANOVA (F_3,34_ = 4.309, *p* < 0.05). The post-hoc analysis further revealed no difference (*p* > 0.05), a trend (*p* < 0.10), and significant effect (*p* < 0.05) in case of no preexposure, one-week pre-exposure and two-weeks pre-exposure, respectively.

We also evaluated alcohol drinking following 0, 1- or 2-week pre-exposure with all the chow controls combined. A mixed-model ANOVA detected a similar relationship between HFD pre-exposure and alcohol drinking with no difference (*p* > 0.05), a trend (*p* < 0.10), and significant effect (*p* < 0.05) in the case of no pre-exposure, one-week pre-exposure and two-weeks pre-exposure, respectively. This relationship was still intact when overall alcohol drinking data in the last 7 days for all the conditions were analyzed by one-way ANOVA (Figure 3D).

**Figure 4:**
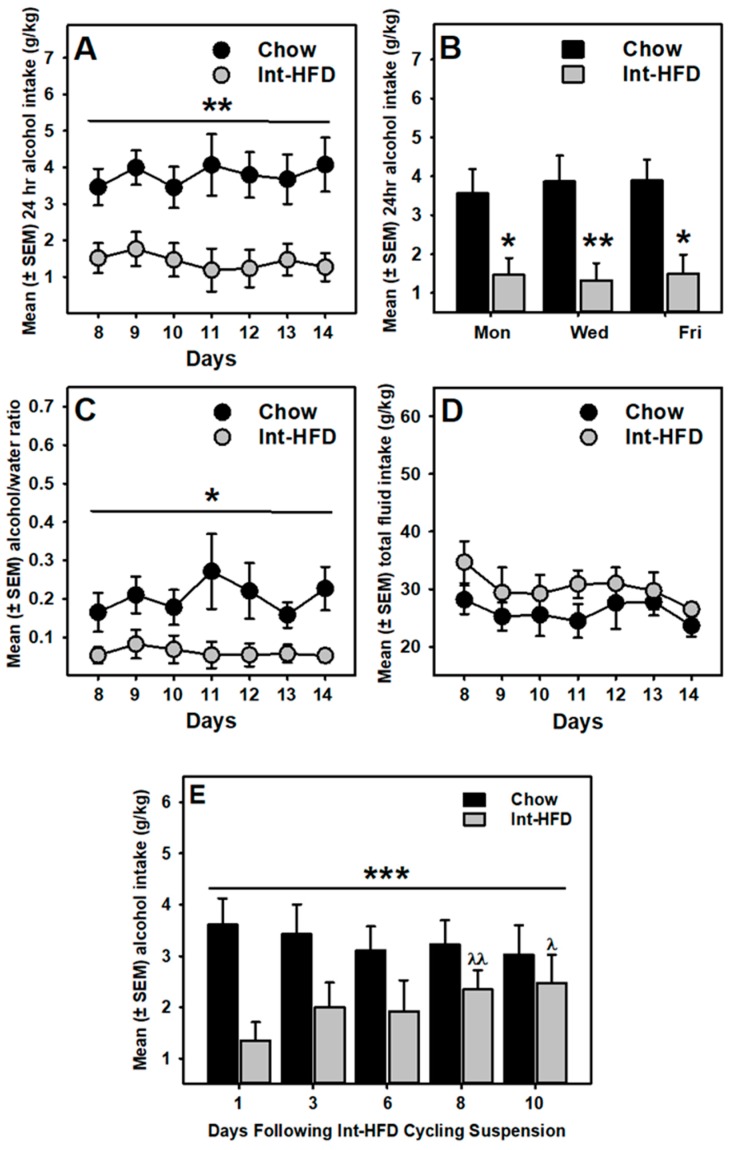
**The intermittent HFD feeding paradigm—induction and regression of reduced alcohol drinking phenotype.** Data represent mean (± SEM) 24 h alcohol consumption (20% *v*/*v*), alcohol preference and total fluid intake for both chow control and Int-HFD group during the last 7 days of testing. (**A**) Reduced alcohol drinking was statistically significant in the second half of testing sessions, including Mondays (**B**). * *p* < 0.05 and ** *p* < 0.01 compared to chow controls. (**C**) Alcohol/water preference was also attenuated in the Int-HFD group, whereas the total fluid intake (**D**) was not significantly different between groups. * *p* < 0.05 main effect of intermittent HFD exposure. (**E**) Data represent mean (± SEM) 24 h alcohol intake for 10 days after the intermittent HFD cycling was suspended. While the chow controls maintained a stable alcohol intake, alcohol intake gradually increased in the Int-HFD group over the testing period. A significant increase in alcohol consumption by Int-HFD group was observed on the eighth and the 10th days, compared to the first day. *** *p* < 0.001 diet×time interaction.

We further assessed alcohol drinking data in rats receiving two weeks of pre-exposure of intermittent HFD exposure to evaluate the induction of reduced alcohol drinking effect. A mixed-model ANOVA identified a trend (*p* = 0.052) towards decrease during the first 7 days, whereas a significant (F_1,10_ = 10.19, *p* < 0.01) decrease in alcohol drinking was observed during the second half of the drinking sessions (Figure 4A). Overall, alcohol drinking was significantly (*p* < 0.05) reduced on all drinking days, including Mondays (Figure 4B). In addition, alcohol preference (Figure 4C) was also significantly (*p* < 0.05) attenuated during these days, whereas no between-group differences were observed in total fluid intake (Figure 4D).

We next examined the impact of suspending intermittent HFD cycling on reduced alcohol drinking behavior. A mixed-model ANOVA identified a significant interaction between time and diet (F_4,40_ = 6.461, *p* < 0.001). Furthermore, alcohol drinking in the chow controls was not significantly different across the testing days but Int-HFD group alcohol intake significantly escalated on the eighth day comparable to chow levels (Figure 4E).

### 3.4. Food Intake and Alcohol Drinking Sessions

In order to evaluate whether HFD withdrawal had any effect on subsequent HFD intake, a one-way repeated measure ANOVA analyzed one-week (two binge-sessions) food intake data before suspension and after HFD reintroduction. However, HFD intake was not statistically different in these sessions, suggesting that HFD withdrawal had no effect on subsequent HFD intake.

**Figure 5:**
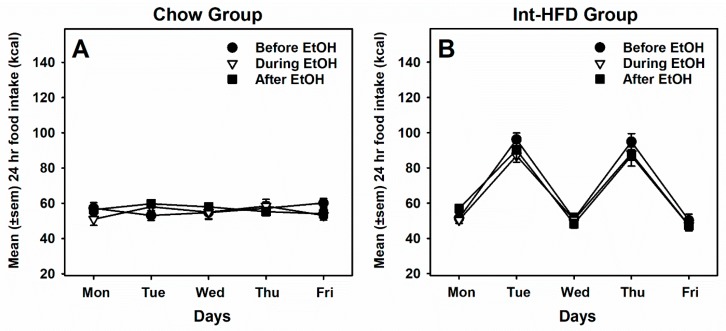
**Daily energy consumption from food before, during and after ethanol testing sessions.** Mean (± SEM) 24 h total food intake (kcal) is presented for chow (**A**) and Int-HFD (**B**) groups before, during and after alcohol drinking testing sessions. Caloric intake was not statistically significant under these three testing conditions in either group.

A two-way repeated measure ANOVA also analyzed average weekly food intake in the chow (Figure 5A) and Int-HFD (Figure 5B) groups before, during and after alcohol testing sessions. However, no statistically significant differences in food intake were observed among any sessions in either group.

### 3.5. Intermittent HFD Exposure and Feeding Peptides

**Figure 6:**
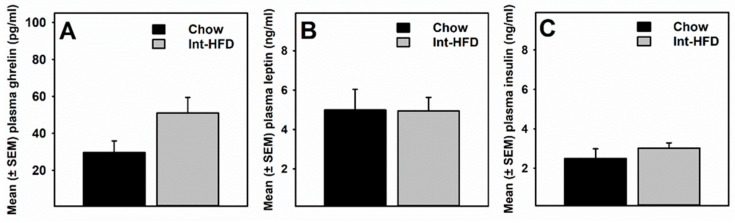
**The intermittent HFD cycling and plasma feeding peptides.** Data represent mean (± SEM) of (**A**) acyl-ghrelin, (**B**) leptin and (**C**) insulin concentrations in the plasma. No statistically significant between-group differences were observed. However, plasma ghrelin concentration was non-significantly elevated (*p* = 0.07) in the Int-HFD group compared to the chow controls.

A *t*-test compared plasma feeding peptide levels in rats displaying a reduced alcohol drinking phenotype following intermittent HFD cycling. Plasma acyl-ghrelin levels were non-significantly (*p* = 0.07) elevated in the Int-HFD group of rats compared to the chow controls (Figure 6A). There were no between-group differences in leptin (Figure 6B) and insulin (Figure 6C) levels. No other feeding peptides were detected.

### 3.6. Intermittent HFD Exposure and Central Neurotransmitter Receptors Gene Expression

**Figure 7:**
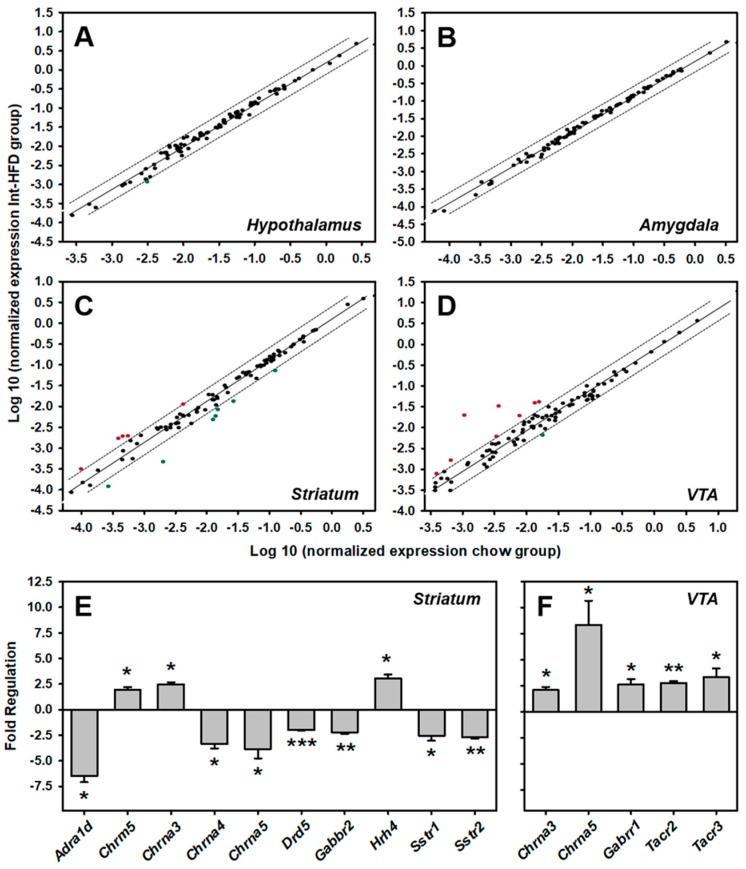
**The intermittent HFD cycling and central neurotransmitter receptors gene expression.** A scatter plot analysis of differential central neurotransmitter receptors genes expression in the hypothalamus (**A**), amygdala (**B**), striatum (**C**) and VTA (**D**). Each dot represents one gene, and the top and bottom genes outside the dotted lines represent a two-fold increase and decrease, respectively. Statistically significant (*p* < 0.05) ≥ two-fold changes in several neurotransmitter receptors gene expression were only observed in the striatum (**E**) and VTA (**F**). * *p* < 0.05, ** *p* < 0.01 and *** *p* < 0.001 compared to the chow controls.

A rat RT^2^ Profiler PCR array examined the expression of 84 neurotransmitter receptors gene expression in the hypothalamus (Figure 7A), amygdala (Figure 7B), striatum (Figure 7C) and ventral tegmental area (VTA) (Figure 7D) of the group of rats that received two weeks of intermittent HFD pre-exposure and compared it with the respective chow controls. A list of genes (Table 1) along with their PCR data is presented for hypothalamus (Table 2), amygdala (Table 3), Striatum (Table 4) and VTA (Table 5). A total of six genes (*Chrm5, Gabra2, Gabrb1, Gabrb3, Gabrg2* and *Gria2*) in Hypothalamus, two genes (*Chrm1* and *Grin2b*) in Amygdala, thirteen genes (*Adra1a, Adra1d, Adra2a, Chrm5, Chrna3, Chrna4, Chrna5, Drd5, Gabbr2, Gabra5, Hrh4, Sstr1* and *Sstr2*) in the Striatum, and eleven genes (*Chrna3, Chrna4, Chrna5, Gabrb1, Gabrr1, Gria3, Htr1a, Htr1b, Sstr2, Tacr2* and *Tacr3*) in VTA were found to be significantly (*p* < 0.05) altered in the Int-HFD group of rats compared to the chow controls. In striatum, a ≥ two-fold statistically significant (*p* < 0.05) increase in three genes (*Chrm5, Chrna3 and Hrh4*) and a decrease in seven genes (*Adra1d, Chrna4, Chrna5, Drd5, Gabbr2, Sstr1 and Sstr2*) was evident (Figure 7E). In VTA, a ≥ two-fold statistically significant (*p* < 0.05) increase was detected in five genes (*Chrna3, Chrna5, Gabrr1, Tacr2* and *Tacr3)* (Figure 7F).

## 4. Discussion

The goal of the present study was to examine the minimum effective duration of an intermittent HFD cycling required to reduce alcohol drinking and its potential neurobiological mechanisms. From this effort, we found that pre-exposure to an intermittent HFD cycling was critical to induce reduced alcohol drinking phenotype, with a significant reduction in alcohol drinking observed following two weeks of pre-exposure. Furthermore, reduced alcohol drinking behavior gradually disappeared following the suspension of intermittent HFD cycling. Moreover, plasma feeding peptides were not significantly different between the Int-HFD group of rats and the chow controls. Finally, greater (significant and ≥two-fold) between-group alterations in the neurotransmitter receptors gene expression levels were found in the striatum and VTA, whereas no such effects were observed in the amygdala and hypothalamus. Overall, these data suggest that pre-exposure and acute availability of an intermittent HFD cycling are critical parameters to reduce alcohol drinking in rats, possibly through mechanisms unlikely involved in maintaining energy homeostasis.

Our original observation of reduced alcohol intake occurred following six weeks of intermittent HFD pre-exposure and alcohol testing in that study occurred while rats were still maintained on intermittent HFD cycling [15]. It was not clear whether a shorter pre-exposure duration (<6 weeks) would be equally effective in reducing alcohol drinking and what would be the impact of suspending intermittent HFD cycling on alcohol drinking. The present study addressed these questions by demonstrating that a pre-exposure of intermittent HFD cycling is necessary to observe reduced alcohol drinking behavior with significantly reduced alcohol drinking observed following two weeks of intermittent HFD pre-exposure (Figure 3 and Figure 4). Interestingly, we observed a pre-exposure-dependent reduction in alcohol drinking with a ~40% reduction (a strong trend) following one week, a ~50% reduction (significant) following two weeks and a ~60% reduction (previous study from our lab [15]) following six weeks of intermittent HFD pre-exposure. Interestingly, the % reduction also correlated with the effect size ~0.16, ~0.44 and ~0.60 following one-week, two-weeks and six-weeks of intermittent HFD exposure, respectively. These data suggest that pre-exposure to intermittent HFD exposure is necessary to attenuate alcohol drinking and with greater pre-exposure, a greater reduction was observed. It also becomes clear that pre-exposure, but not intermittent availability or the diet itself, seems to play a role in attenuating alcohol drinking as rats receiving no intermittent HFD pre-exposure did not display reduced alcohol drinking behavior. Furthermore, alcohol drinking in the chow control group escalated over time, whereas it stayed almost the same in the case of the Int-HFD group, which suggests that intermittent HFD pre-exposure impaired acquisition of alcohol drinking behavior.

Previously, we have also observed a reduction in alcohol drinking when HFD intake was restricted to 2 h/day (which induced a binge-like HFD feeding) either three times a week or every day [14]. However, a recent study reported increased alcohol self-administration following binge-like HFD exposure [18]. The time of intermittent HFD exposure (adolescence in the case of Blanco-Gandia et al. and adult animals in our study) or testing conditions (restricted feeding in the case of Blanco-Gandia et al. and unrestricted chow presence in our study) could be responsible for these discrepancies. It is also important to note here that alcohol testing in the present and previous studies from our lab occurred on the following days of HFD exposure and HFD was never presented concurrently with alcohol on the same day [14,15], whereas animals continued to binge-eat during self-administration in Blanco-Gandia et al. [18]. Nevertheless, the present study identified a feeding paradigm that effectively reduces alcohol drinking following a short period of intermittent HFD cycling and helps to decipher some of the critical parameters needed to understand the impact of dietary manipulations on alcohol drinking behavior.

It is also important to note that reduced alcohol drinking behavior was not only observed on Wednesdays and Fridays (testing days immediately following HFD access days) but also on Mondays (three days following the last HFD access session) (Figure 4B), suggesting that caloric overload one day prior to alcohol testing is not likely to reduce alcohol drinking in the present study. Furthermore, alcohol preference was also significantly attenuated in the Int-HFD group compared to controls, whereas total fluid intake was unaffected (Figure 4C–D). When tested under similar conditions, blood alcohol concentrations did not differ between groups, indicating that alcohol metabolism or absorption were not affected following Int-HFD exposure [15].

In the previous and the present study from our lab, intermittent HFD cycling continued during the alcohol testing period (see Figure 1B). Therefore, it was equally important to evaluate the impact of the suspension of intermittent HFD cycling on alcohol drinking. Therefore, we evaluated alcohol drinking following the suspension of intermittent HFD cycling. Under these conditions, reduced alcohol drinking behavior gradually disappeared within a week following the termination of intermittent HFD cycling (Figure 4E). Although alcohol drinking increased significantly following HFD removal, it never reached the levels observed in the chow-controls. We have previously observed that the Int-HFD exposed group, under similar conditions, continued to display a non-significant but lower alcohol intake over 24 days and alcohol drinking was never escalated compared to the chow controls (unpublished observation).

To further determine the mechanisms responsible for the diet-induced reduction in alcohol drinking, we examined plasma feeding peptides and central neurotransmitters genes expression analysis in behaviorally characterized rats that displayed reduced alcohol drinking behavior. Since alcohol drinking was significantly reduced in rats receiving two-weeks intermittent HFD exposure, these measures occurred in this group of rats only.

It is becoming clear that neuroendocrine feeding peptides can interact with the brain reward circuitry to regulate the intake and reinforcing properties of both food and alcohol [19]. Therefore, we examined a panel of these gastrointestinal peptides using a multiplexing assay. While the acyl-ghrelin concentration was slightly elevated in the Int-HFD group of rats compared to the chow controls, no statistically significant between-group differences existed (Figure 6). Ghrelin, a gut-derived feeding peptide, has been positively linked to alcohol intake and is elevated in abstinent alcoholics [19,20,21]. However, in the present study, acyl-ghrelin was non-significantly elevated in the Int-HFD groups of rats which were drinking less alcohol. This increase in ghrelin is consistent with our previous study in which ghrelin was assessed under similar conditions [15], suggesting that peripheral ghrelin could be less likely to be involved in regulating alcohol drinking at least in the present paradigm. This contention is in agreement with a recent study demonstrating the importance of the central ghrelin receptor (GHSR) activity instead of peripheral ghrelin levels, as a regulator of alcohol intake [22]. However, we did not find alterations in the GHSR gene expression in the ventral striatum in the rats exposed to a similar intermittent HFD paradigm either [15].

Finally, we examined the expression of over 84 central neurotransmitters genes in the brain regions implicated in the regulation of energy balance or reinforced behavior using a PCR array. As discussed below, selective alterations in several gene expression were registered in the striatum and VTA, whereas no significant changes were observed in the amygdala and hypothalamus of rats receiving two-weeks of HFD pre-exposure.

In the striatum, alpha1 adrenergic receptors (α-1AR) are primarily located in the axon terminals and are capable of regulating multiple neurotransmissions, including dopamine [23,24,25]. Studies have shown that α-1ARs are capable of stimulating dopamine release in the striatal area and local administration of prazosin (α-1AR antagonist) reduces dopamine neurotransmission [26]. Interestingly, prazosin treatment has been shown to reduce alcohol drinking in alcohol-preferring rats [27] and facilitate abstinence in human alcoholics [28] and its effects on alcohol intake are centrally mediated. Adrenergic, alpha-1D-, receptor (*Adra1d*) receptor gene expression was reduced ~six-fold in the Int-HFD group of rats. Therefore, reduced dopaminergic neurotransmission, as a result of decreased α-1AR tone in the striatal region of intermittent HFD exposed rats, could explain reduced alcohol drinking. Further studies are needed to address this possibility.

Studies have reported reduced dopaminergic turnover, independent of obesity, following HFD feeding [29]. We also observed reduced expression of Dopamine receptor D5 in the striatal area. Dopamine (DA) mediates its activity via D1-like (D1 and D5) and D2-like (D2, D3, D4) receptors which are widely distributed in the CNS [30]. A recent study demonstrated that D1-like, but not D2-like receptor blockade, in the striatal region impaired alcohol-induced conditioned place preference [31] and reduced alcohol-seeking behavior by modulating the neuronal activation in response to alcohol-associated cues [32]. While the involvement of the D5 receptor in regulating alcohol drinking or reward is less clear, a study has shown that D5 receptors are present and depolarize both cholinergic and GABAergic striatal interneurons following activation by DA and thereby are capable of modulating striatal GABAergic medium spiny projection neurons activity [33]. Considering that alcohol in acute intoxicating concentrations can alter both of these inter-neuronal activities and resultant projection neurons [34], reduced D5 receptor gene expression following intermittent HFD exposure, as seen in the present case, could blunt alcohol-mediated activities within this region, a contention that needs further investigation.

Striatum not only receives inputs from histaminergic neurons from the hypothalamus but also expresses a high density of histamine receptors, including H4 receptor [35,36]. These histamine receptors are positioned to differentially modulate striatal neurotransmission by impacting various excitatory and inhibitory neurocircuits [37]. We found significantly increased H4 gene expression in the striatum of Int-HFD group of rats. However, the functional significance of these findings remains unclear as very little is known about the newly discovered H4 receptors and future studies will shed light on this.

Another receptor type that was impacted by intermittent HFD exposure in the present study was somatostatin receptor (SstR). Striatum displays high SstR density [38] and a functional linkage between the somatostatinergic and dopaminergic neurocircuitry has been registered [39]. Furthermore, SstRs stimulation has been shown to stimulate striatal DA release in rodents [40]. Therefore, reduced Sstrs expression and resultant modulations of striatal DA neurotransmission could be linked to the reduced alcohol drinking in the Int-HFD exposed group of rats which would require further investigation.

Cholinergic receptors are implicated in complex controls of striatal neurocircuitry, including dopaminergic neurotransmission [41,42,43]. Furthermore, acute alcohol administration has been shown to upregulate nicotinic acetylcholine receptors (alpha 4) within the striatum [44] and reduced striatal nicotinic acetylcholine receptors (alpha 4 and 7) density has been reported in alcohol-preferring rats [45]. While chronic alcohol exposure has not been shown to alter muscarinic binding in rat striatum [46], muscarinic acetylcholine receptors (M5) have been shown to regulate DAergic neurotransmission in nucleus accumbens and alcohol drinking and seeking in rodents [47,48]. Intermittent HFD exposure in the present study regulated some of these muscarinic and nicotinic acetylcholine receptors (Figure 7E). It is important to mention here that both dorsal and ventral striatum were pooled in the present study, collectively referred to as the striatum. Since different receptor types could mediate region-specific (dorsal vs. ventral) alterations in the DAergic neurotransmission and ultimately striatal output, future research is needed to site-specifically assess the alterations in these neurotransmitter receptors following intermittent HFD exposure.

Ethanol activates VTA DAergic neurons by interacting with the nicotinic acetylcholine receptors (nAChRs) and thereby releasing DA in the nucleus accumbens and this activation could be reversed by VTA nAChRs blockade [49]. While several nicotinic subunits, including α3 and α5, are present in the VTA DAergic neurons [50], it is relatively unclear which nAChRs subunits mediate ethanol induced DAergic neuronal activation (for details see [51]). In this regard, high affinity α3β4 partial agonists have been shown to reduce ethanol consumption and self-administration [52]. In VTA, we found that nicotinic acetylcholine receptors (α3 and α5) gene expression was upregulated (Figure 7F) in the Int-HFD group of rats. Therefore, it is possible that Int-HFD exposure moderately increases overall VTA dopaminergic tone, making Int-HFD exposed rats more sensitive to the intoxicating effects of alcohol and thereby reducing alcohol drinking, a contention that needs further investigation.

We also found that gamma-aminobutyric acid (GABA) receptor (ρ1) gene expression was upregulated in the VTA of Int-HFD group of rats. While it is known that ethanol enhances GABA-A receptor function, this pentameric structure is extensively heterogeneous due to several subunits (α, β, γ, δ, ε, θ, π and ρ). Evidence exists that gamma-aminobutyric acid (GABA) receptor (ρ1), which is encoded by the *GABRR1* gene, is inhibited by ethanol [53]. Interestingly, genetic deletion of *GABRR1* potentiated sedative motor effects of ethanol in mice [54] and SNPs in *GABRR1* and *GABRR2* genes are associated with alcohol use disorder [55]. Future studies are needed to evaluate the significance of increased GABRR1 following intermittent HFD cycling.

Finally, we also observed increased expression of Tacr2 and Tacr3 genes which encode for tachykinin receptors 2 and 3, respectively. Interestingly, stimulation of these receptors has been shown to reduce alcohol drinking, without impacting food or water intake [56] and SNPs in Tacr3 gene are linked in alcohol and cocaine dependence [57]. Based on this, it could be that intermittent HFD exposure-induced reduction in alcohol drinking is linked to increased tachykinin receptor activity but requires further experimental validation.

It is important to note here that these gene expression analyses were conducted in the brains of rats not only showing increased HFD intake but also reduced alcohol drinking compared to the chow controls. While this could be a strength of the present study, as these analyses occurred in behaviorally characterized rats, an approach successfully used in the past by our group [14,15,17,58], a potential limitation of this approach could be that contribution of HFD alone in producing these changes cannot be easily deciphered. Although brains were collected ~9–12 days after stopping alcohol drinking sessions while rats were still on intermittent HFD cycling, future studies in which rats receive identical Int-HFD treatment without alcohol exposure would be needed to precisely understand the impact of intermittent HFD exposure on these genes.

Overall, the amygdala and hypothalamus neurotransmitter receptors gene expression were least impacted following intermittent HFD exposure, which speaks against the involvement of these brain regions in reducing alcohol drinking following intermittent HFD exposure. The hypothalamus is the energy regulation center in the brain that modulates feeding behavior by integrating multiple hormonal and neuronal signals originating from complex interactions of multiple feeding regulators (e.g., insulin, leptin and ghrelin etc.). Our PCR array data in the hypothalamus are also in agreement with the plasma feeding peptide data, suggesting that homeostatic mechanisms were less likely to be engaged as a result of our intermittent HFD exposure paradigm to account for reduced alcohol drinking behavior in the Int-HFD group. Instead, greater alterations in the genetic regulation were seen in the brain reward circuitry of the intermittent HFD group compared to the chow controls. Since palatable foods are capable of activating brain reward circuitry, similarly to drugs of abuse [12,59,60,61], reduced alcohol drinking in the present study could reflect the adjustment in the brain reward circuitry following intermittent HFD exposure, a contention that requires further evaluation.

The present study warrants further investigations to evaluate the benefits of nutritional contingency in alcoholism. However, it is too early to extend clinically appropriate dietary recommendations for the treatment of alcoholism as several critical parameters of such dietary intervention are unknown. For example, it is unclear if a diet high in fat is necessary to trigger reduced alcohol drinking behavior or if a diet high in sugar would induce the same effect. While a recent study showed a reduction of alcohol withdrawal syndrome following a high fat/ketogenic diet intake [62], studies exist demonstrating that excessive consumption of palatable/sweet food could curb alcohol drinking behavior in human alcoholics [10,11] which also agrees with the Alcoholics Anonymous recommendation of eating more sweet/palatable food to reduce alcohol craving [63]. These data collectively suggest that palatable food may compete with propensity to drink alcohol as both are capable of interacting with the brain reward circuitry. It is also important to consider the negative health consequences associated with excessive consumption of palatable foods. Pertinent to this, a previous study from our lab demonstrate and even 2 h of HFD exposure every third day could reduce alcohol drinking, a paradigm which does not impact body weight or fat [14]. Furthermore, dietary exposure of such palatable diets may be restricted to the early detoxification period and could be gradually reduced to minimize the negative consequences associated with these diets. On the other hand, since alcoholics are malnourished and have lower BMIs, a micro and macro-nutrient rich diet could not only reduce alcohol drinking but also may improve nutritional status over treatment duration, contributing to treatment success.

## 5. Conclusions

In conclusion, the present study identified key parameters that allow intermittent HFD cycling to reduce alcohol drinking, an effect possibly mediated by alterations in the brain reward circuitry. These data have important clinical implications as an altered nutritional status has been frequently documented in patients with AUD, which could contribute to escalated alcohol consumption and behavioral impairments commonly observed in AUD [6,9,64,65,66]. In this context, it is clear that contingency management (providing a tangible reward for drug abstinence) is an effective method for the management of substance use and alcohol use disorders [67]. A very recent study reported that an alternative palatable food reward could maintain prolonged abstinence from methamphetamine [68]. It is possible that improving nutritional status during abstinence would not only compensate for general malnutrition in alcoholics but could also ameliorate some of the adverse symptoms observed in alcohol withdrawal, thereby enhancing prospects of recovery. Therefore, future studies testing if a similar feeding paradigm would be effective in reducing alcohol drinking in the models of AUD are needed to functionally assess the importance of such dietary interventions. Importantly, these data highlight some of the critical behavioral and genetic components that can be used to refine nutritional contingency in the management of AUD.

## Figures and Tables

**Table 1:** A panel of genes examined using rat neurotransmitter receptors RT^2^ Profiler PCR array.

**Adrenergic Receptors**	**Genes**
Alpha Adrenergic Receptors	*Adra1a, Adra1d, Adra2a*
Beta Adrenergic Receptors	*Adrb2, Adrb3*
**Cholinergic Receptors**	
Muscarinic Receptors	*Chrm1, Chrm4, Chrm5*
Nicotinic Receptors	*Chrna3, Chrna4, Chrna5, Chrna6, Chrna7, Chrne*
**Dopaminergic Receptors**	*Drd1, Drd2, Drd5*
**GABAergic Receptors**	
GABAC Receptors	*Gabrr1, Gabrr2*
GABAB Receptors	*Gabbr1, Gabbr2*
GABAA Receptors	*Gabra1, Gabra2, Gabra4, Gabra5, Gabra6, Gabrb1, Gabrb3, Gabrd, Gabre, Gabrg1, Gabrg2, Gabrg3, Gabrq*
**Glutamate Receptors**	
AMPA Receptors	*Gria1, Gria2, Gria3*
Kainate Receptors	*Grik1, Grik2, Grik4, Grik5*
NMDA Receptors	*Grin1, Grin2a, Grin2b, Grin2c*
Metabotropic Receptors	*Grm1, Grm3, Grm4, Grm5, Grm6, Grm7, Grm8*
**Serotonin Receptors**	*Htr1a, Htr1b, Htr1d, Htr1f, Htr2a, Htr2c, Htr3a, Htr4, Htr7*
**Other Neurotransmitter Receptors**	
Vasopressin Receptor	*Avpr1a, Avpr1b*
Histamine Receptors	*Hrh1, Hrh4*
Neuropeptide Y Receptors	*Npy2r, Npy5r*
Somatostatin Receptors	*Sstr1, Sstr2, Sstr4*
Substance P (Neurokinin) Receptors	*Tacr1, Tacr2, Tacr3*
Other Neurotransmitter Receptors	*Brs3, Cckbr, Cnr1, Gcgr, Grpr, Hcrtr2, Ntsr2, Oxtr, Prokr2 (Gpr73l1), Sctr, Tspo (Bzrp)*

**Table 2:** Intermittent HFD exposure and rat neurotransmitter receptors gene expression in the hypothalamus using the RT^2^ Profiler PCR Array. The bold underlined text highlights statistically significant changes compared to the chow controls.

#	Unigene	Refseq	Symbol	Description	Fold Regulation	*p* Value
1	Rn.9991	NM_017191	*Adra1a*	Adrenergic, alpha-1A-, receptor	−1.0	0.877
2	Rn.11314	NM_024483	*Adra1d*	Adrenergic, alpha-1D-, receptor	−1.8	0.236
3	Rn.170171	NM_012739	*Adra2a*	Adrenergic, alpha-2A-, receptor	−1.0	0.887
4	Rn.10206	NM_012492	*Adrb2*	Adrenergic, beta-2-, receptor, surface	−1.1	0.649
5	Rn.32282	NM_053019	*Avpr1a*	Arginine vasopressin receptor 1A	1.1	0.403
6	Rn.10096	NM_017205	*Avpr1b*	Arginine vasopressin receptor 1B	−1.3	0.455
7	Rn.86415	NM_152845	*Brs3*	Bombesin-like receptor 3	1.4	0.230
8	Rn.90997	NM_013165	*Cckbr*	Cholecystokinin B receptor	1.5	0.341
9	Rn.119395	NM_080773	*Chrm1*	Cholinergic receptor, muscarinic 1	1.9	0.050
10	Rn.10676	NM_031547	*Chrm4*	Cholinergic receptor, muscarinic 4	1.7	0.005
11	Rn.44293	NM_017362	*Chrm5*	Cholinergic receptor, muscarinic 5	1.2	0.296
12	Rn.10996	NM_052805	*Chrna3*	Cholinergic receptor, nicotinic, alpha 3	−1.1	0.548
13	Rn.9697	NM_024354	*Chrna4*	Cholinergic receptor, nicotinic, alpha 4	−1.2	0.618
14	Rn.40125	NM_017078	*Chrna5*	Cholinergic receptor, nicotinic, alpha 5	−1.6	0.588
15	Rn.9696	NM_057184	*Chrna6*	Cholinergic receptor, nicotinic, alpha 6	−1.4	0.573
16	Rn.9698	NM_012832	*Chrna7*	Cholinergic receptor, nicotinic, alpha 7	1.3	0.283
17	Rn.10301	NM_017194	*Chrne*	Cholinergic receptor, nicotinic, epsilon	1.1	0.405
18	Rn.89774	NM_012784	*Cnr1*	Cannabinoid receptor 1 (brain)	1.1	0.358
19	Rn.24039	NM_012546	*Drd1*	Dopamine receptor D1A	1.6	0.066
20	Rn.87299	NM_012547	*Drd2*	Dopamine receptor D2	−1.1	0.672
21	Rn.138110	NM_012768	*Drd5*	Dopamine receptor D5	1.1	0.413
22	Rn.30059	NM_031028	*Gabbr1*	Gamma-aminobutyric acid (GABA) B receptor 1	1.1	0.498
23	Rn.162814	NM_031802	*Gabbr2*	Gamma-aminobutyric acid (GABA) B receptor 2	−1.1	0.525
24	Rn.28463	NM_183326	*Gabra1*	Gamma-aminobutyric acid (GABA) A receptor, alpha 1	−1.1	0.443
25	Rn.48180	NM_001135779	*Gabra2*	Gamma-aminobutyric acid (GABA-A) receptor, subunit alpha 2	1.3	0.020
26	Rn.81205	NM_080587	*Gabra4*	Gamma-aminobutyric acid (GABA) A receptor, alpha 4	1.1	0.759
27	Rn.10368	NM_017295	*Gabra5*	Gamma-aminobutyric acid (GABA) A receptor, alpha 5	1.2	0.254
28	Rn.29890	NM_021841	*Gabra6*	Gamma-aminobutyric acid (GABA) A receptor, alpha 6	−2.2	0.359
29	Rn.207157	NM_012956	*Gabrb1*	Gamma-aminobutyric acid (GABA) A receptor, beta 1	1.3	0.010
30	Rn.233948	NM_017065	*Gabrb3*	Gamma-aminobutyric acid (GABA) A receptor, beta 3	1.3	0.045
31	Rn.10927	NM_017289	*Gabrd*	Gamma-aminobutyric acid (GABA) A receptor, delta	−1.3	0.676
32	Rn.54455	NM_023091	*Gabre*	Gamma-aminobutyric acid (GABA) A receptor, epsilon	1.3	0.357
33	Rn.10366	NM_080586	*Gabrg1*	Gamma-aminobutyric acid (GABA) A receptor, gamma 1	1.1	0.140
34	Rn.230132	NM_183327	*Gabrg2*	Gamma-aminobutyric acid (GABA) A receptor, gamma 2	1.2	0.040
35	Rn.10369	NM_024370	*Gabrg3*	Gamma-aminobutyric acid (GABA) A receptor, gamma 3	1.1	0.554
36	Rn.81067	NM_031733	*Gabrq*	Gamma-aminobutyric acid (GABA) receptor, theta	1.4	0.098
37	Rn.33552	NM_017291	*Gabrr1*	Gamma-aminobutyric acid (GABA) receptor, rho 1	−1.1	0.617
38	Rn.48659	NM_017292	*Gabrr2*	Gamma-aminobutyric acid (GABA) receptor, rho 2	−1.2	0.325
39	Rn.11225	NM_172092	*Gcgr*	Glucagon receptor	−1.1	0.666
40	Rn.29971	NM_031608	*Gria1*	Glutamate receptor, ionotropic, AMPA 1	1.2	0.106
41	Rn.91361	NM_017261	*Gria2*	Glutamate receptor, ionotropic, AMPA 2	1.1	0.015
42	Rn.74049	NM_032990	*Gria3*	Glutamate receptor, ionotrophic, AMPA 3	−1.1	0.721
43	Rn.10449	NM_017241	*Grik1*	Glutamate receptor, ionotropic, kainate 1	1.0	0.702
44	Rn.87696	NM_019309	*Grik2*	Glutamate receptor, ionotropic, kainate 2	1.1	0.082
45	Rn.10049	NM_012572	*Grik4*	Glutamate receptor, ionotropic, kainate 4	−1.0	0.670
46	Rn.74042	NM_031508	*Grik5*	Glutamate receptor, ionotropic, kainate 5	1.2	0.059
47	Rn.9840	NM_017010	*Grin1*	Glutamate receptor, ionotropic, N-methyl D-aspartate 1	1.1	0.254
48	Rn.9710	NM_012573	*Grin2a*	Glutamate receptor, ionotropic, N-methyl D-aspartate 2A	−1.0	0.888
49	Rn.9711	NM_012574	*Grin2b*	Glutamate receptor, ionotropic, N-methyl D-aspartate 2B	1.1	0.342
50	Rn.9709	NM_012575	*Grin2c*	Glutamate receptor, ionotropic, N-methyl D-aspartate 2C	1.0	0.971
51	Rn.87787	NM_017011	*Grm1*	Glutamate receptor, metabotropic 1	1.1	0.073
52	Rn.41715	NM_001105712	*Grm3*	Glutamate receptor, metabotropic 3	−1.5	0.147
53	Rn.89046	NM_022666	*Grm4*	Glutamate receptor, metabotropic 4	−1.1	0.724
54	Rn.29972	NM_017012	*Grm5*	Glutamate receptor, metabotropic 5	1.1	0.356
55	Rn.44615	NM_022920	*Grm6*	Glutamate receptor, metabotropic 6	−1.1	0.753
56	Rn.10409	NM_031040	*Grm7*	Glutamate receptor, metabotropic 7	1.2	0.055
57	Rn.44420	NM_022202	*Grm8*	Glutamate receptor, metabotropic 8	−1.1	0.312
58	Rn.10316	NM_012706	*Grpr*	Gastrin releasing peptide receptor	1.6	0.136
59	Rn.9893	NM_013074	*Hcrtr2*	Hypocretin (orexin) receptor 2	1.3	0.159
60	Rn.81032	NM_017018	*Hrh1*	Histamine receptor H 1	1.3	0.272
61	Rn.162272	NM_131909	*Hrh4*	Histamine receptor H4	−1.7	0.428
62	Rn.44486	NM_012585	*Htr1a*	5-hydroxytryptamine (serotonin) receptor 1A	−1.1	0.723
63	Rn.138109	NM_022225	*Htr1b*	5-hydroxytryptamine (serotonin) receptor 1B	1.0	0.796
64	Rn.34834	NM_012852	*Htr1d*	5-Hydroxytryptamine (serotonin) receptor 1D	1.0	0.893
65	Rn.44301	NM_021857	*Htr1f*	5-hydroxytryptamine (serotonin) receptor 1F	−1.1	0.630
66	Rn.10294	NM_017254	*Htr2a*	5-hydroxytryptamine (serotonin) receptor 2A	1.2	0.682
67	Rn.9935	NM_012765	*Htr2c*	5-hydroxytryptamine (serotonin) receptor 2C	−1.0	0.949
68	Rn.55109	NM_024394	*Htr3a*	5-hydroxytryptamine (serotonin) receptor 3a	−1.2	0.444
69	Rn.10094	NM_012853	*Htr4*	5-hydroxytryptamine (serotonin) receptor 4	1.1	0.051
70	Rn.87132	NM_022938	*Htr7*	5-hydroxytryptamine (serotonin) receptor 7	1.0	0.409
71	Rn.64505	NM_023968	*Npy2r*	Neuropeptide Y receptor Y2	1.7	0.108
72	Rn.10532	NM_012869	*Npy5r*	Neuropeptide Y receptor Y5	1.1	0.691
73	Rn.127792	NM_022695	*Ntsr2*	Neurotensin receptor 2	1.0	0.849
74	Rn.6841	NM_012871	*Oxtr*	Oxytocin receptor	1.4	0.118
75	Rn.82760	NM_138978	*Prokr2*	Prokineticin receptor 2	1.4	0.234
76	Rn.32256	NM_031115	*Sctr*	Secretin receptor	−1.2	0.630
77	Rn.42915	NM_012719	*Sstr1*	Somatostatin receptor 1	1.2	0.258
78	Rn.9929	NM_019348	*Sstr2*	Somatostatin receptor 2	1.0	0.762
79	Rn.9936	NM_013036	*Sstr4*	Somatostatin receptor 4	1.1	0.417
80	Rn.89609	NM_012667	*Tacr1*	Tachykinin receptor 1	1.1	0.215
81	Rn.202846	NM_080768	*Tacr2*	Tachykinin receptor 2	−1.8	0.333
82	Rn.9702	NM_017053	*Tacr3*	Tachykinin receptor 3	1.2	0.286
83	Rn.1820	NM_012515	*Tspo*	Translocator protein	1.1	0.489

**Table 3:** Intermittent HFD exposure and rat neurotransmitter receptors gene expression in the amygdala using the RT^2^ Profiler PCR Array. The bold underlined text highlights statistically significant changes compared to the chow controls.

#	Unigene	Refseq	Symbol	Description	Fold Regulation	*p* Value
1	Rn.9991	NM_017191	*Adra1a*	Adrenergic, alpha-1A-, receptor	1.2	0.228
2	Rn.11314	NM_024483	*Adra1d*	Adrenergic, alpha-1D-, receptor	1.2	0.889
3	Rn.170171	NM_012739	*Adra2a*	Adrenergic, alpha-2A-, receptor	1.1	0.931
4	Rn.10206	NM_012492	*Adrb2*	Adrenergic, beta-2-, receptor, surface	−1.2	0.208
5	Rn.10100	NM_013108	*Adrb3*	Adrenergic, beta-3-, receptor	−1.4	0.182
6	Rn.32282	NM_053019	*Avpr1a*	Arginine vasopressin receptor 1A	−1.1	0.613
7	Rn.10096	NM_017205	*Avpr1b*	Arginine vasopressin receptor 1B	1.0	0.865
8	Rn.86415	NM_152845	*Brs3*	Bombesin-like receptor 3	−1.4	0.597
9	Rn.90997	NM_013165	*Cckbr*	Cholecystokinin B receptor	1.1	0.427
10	Rn.119395	NM_080773	*Chrm1*	Cholinergic receptor, muscarinic 1	1.1	0.041
11	Rn.10676	NM_031547	*Chrm4*	Cholinergic receptor, muscarinic 4	−1.0	0.857
12	Rn.44293	NM_017362	*Chrm5*	Cholinergic receptor, muscarinic 5	−1.2	0.746
13	Rn.10996	NM_052805	*Chrna3*	Cholinergic receptor, nicotinic, alpha 3	1.0	0.755
14	Rn.9697	NM_024354	*Chrna4*	Cholinergic receptor, nicotinic, alpha 4	1.1	0.287
15	Rn.40125	NM_017078	*Chrna5*	Cholinergic receptor, nicotinic, alpha 5	1.2	0.746
16	Rn.9696	NM_057184	*Chrna6*	Cholinergic receptor, nicotinic, alpha 6	1.1	0.347
17	Rn.9698	NM_012832	*Chrna7*	Cholinergic receptor, nicotinic, alpha 7	1.1	0.482
18	Rn.10301	NM_017194	*Chrne*	Cholinergic receptor, nicotinic, epsilon	1.1	0.172
19	Rn.89774	NM_012784	*Cnr1*	Cannabinoid receptor 1 (brain)	1.2	0.286
20	Rn.24039	NM_012546	*Drd1*	Dopamine receptor D1A	1.1	0.953
21	Rn.87299	NM_012547	*Drd2*	Dopamine receptor D2	−1.0	0.852
22	Rn.138110	NM_012768	*Drd5*	Dopamine receptor D5	1.1	0.797
23	Rn.30059	NM_031028	*Gabbr1*	Gamma-aminobutyric acid (GABA) B receptor 1	1.0	0.662
24	Rn.162814	NM_031802	*Gabbr2*	Gamma-aminobutyric acid (GABA) B receptor 2	1.2	0.210
25	Rn.28463	NM_183326	*Gabra1*	Gamma-aminobutyric acid (GABA) A receptor, alpha 1	1.1	0.646
26	Rn.48180	NM_001135779	*Gabra2*	Gamma-aminobutyric acid (GABA-A) receptor, subunit alpha 2	1.1	0.741
27	Rn.81205	NM_080587	*Gabra4*	Gamma-aminobutyric acid (GABA) A receptor, alpha 4	−1.0	0.961
28	Rn.10368	NM_017295	*Gabra5*	Gamma-aminobutyric acid (GABA) A receptor, alpha 5	1.1	0.460
29	Rn.29890	NM_021841	*Gabra6*	Gamma-aminobutyric acid (GABA) A receptor, alpha 6	−1.6	0.377
30	Rn.207157	NM_012956	*Gabrb1*	Gamma-aminobutyric acid (GABA) A receptor, beta 1	1.2	0.417
31	Rn.233948	NM_017065	*Gabrb3*	Gamma-aminobutyric acid (GABA) A receptor, beta 3	1.1	0.090
32	Rn.10927	NM_017289	*Gabrd*	Gamma-aminobutyric acid (GABA) A receptor, delta	−1.2	0.440
33	Rn.54455	NM_023091	*Gabre*	Gamma-aminobutyric acid (GABA) A receptor, epsilon	1.2	0.619
34	Rn.10366	NM_080586	*Gabrg1*	Gamma-aminobutyric acid (GABA) A receptor, gamma 1	1.0	0.952
35	Rn.230132	NM_183327	*Gabrg2*	Gamma-aminobutyric acid (GABA) A receptor, gamma 2	1.0	0.631
36	Rn.10369	NM_024370	*Gabrg3*	Gamma-aminobutyric acid (GABA) A receptor, gamma 3	1.2	0.165
37	Rn.81067	NM_031733	*Gabrq*	Gamma-aminobutyric acid (GABA) receptor, theta	1.0	0.920
38	Rn.33552	NM_017291	*Gabrr1*	Gamma-aminobutyric acid (GABA) receptor, rho 1	1.1	0.728
39	Rn.48659	NM_017292	*Gabrr2*	Gamma-aminobutyric acid (GABA) receptor, rho 2	−1.3	0.280
40	Rn.11225	NM_172092	*Gcgr*	Glucagon receptor	−1.6	0.581
41	Rn.29971	NM_031608	*Gria1*	Glutamate receptor, ionotropic, AMPA 1	1.1	0.426
42	Rn.91361	NM_017261	*Gria2*	Glutamate receptor, ionotropic, AMPA 2	1.0	0.587
43	Rn.74049	NM_032990	*Gria3*	Glutamate receptor, ionotrophic, AMPA 3	1.1	0.475
44	Rn.10449	NM_017241	*Grik1*	Glutamate receptor, ionotropic, kainate 1	−1.0	0.677
45	Rn.87696	NM_019309	*Grik2*	Glutamate receptor, ionotropic, kainate 2	1.2	0.118
46	Rn.10049	NM_012572	*Grik4*	Glutamate receptor, ionotropic, kainate 4	−1.0	0.882
47	Rn.74042	NM_031508	*Grik5*	Glutamate receptor, ionotropic, kainate 5	1.0	0.886
48	Rn.9840	NM_017010	*Grin1*	Glutamate receptor, ionotropic, N-methyl D-aspartate 1	1.1	0.468
49	Rn.9710	NM_012573	*Grin2a*	Glutamate receptor, ionotropic, N-methyl D-aspartate 2A	1.1	0.372
50	Rn.9711	NM_012574	*Grin2b*	Glutamate receptor, ionotropic, N-methyl D-aspartate 2B	1.2	0.014
51	Rn.9709	NM_012575	*Grin2c*	Glutamate receptor, ionotropic, N-methyl D-aspartate 2C	−1.0	0.888
52	Rn.87787	NM_017011	*Grm1*	Glutamate receptor, metabotropic 1	1.1	0.280
53	Rn.41715	NM_001105712	*Grm3*	Glutamate receptor, metabotropic 3	1.1	0.485
54	Rn.89046	NM_022666	*Grm4*	Glutamate receptor, metabotropic 4	1.1	0.301
55	Rn.29972	NM_017012	*Grm5*	Glutamate receptor, metabotropic 5	1.1	0.164
56	Rn.44615	NM_022920	*Grm6*	Glutamate receptor, metabotropic 6	1.3	0.248
57	Rn.10409	NM_031040	*Grm7*	Glutamate receptor, metabotropic 7	1.2	0.079
58	Rn.44420	NM_022202	*Grm8*	Glutamate receptor, metabotropic 8	1.3	0.494
59	Rn.10316	NM_012706	*Grpr*	Gastrin releasing peptide receptor	1.4	0.356
60	Rn.9893	NM_013074	*Hcrtr2*	Hypocretin (orexin) receptor 2	−1.1	0.733
61	Rn.81032	NM_017018	*Hrh1*	Histamine receptor H 1	−1.3	0.137
62	Rn.162272	NM_131909	*Hrh4*	Histamine receptor H4	−1.2	0.654
63	Rn.44486	NM_012585	*Htr1a*	5-hydroxytryptamine (serotonin) receptor 1A	1.1	0.575
64	Rn.138109	NM_022225	*Htr1b*	5-hydroxytryptamine (serotonin) receptor 1B	1.2	0.360
65	Rn.34834	NM_012852	*Htr1d*	5-Hydroxytryptamine (serotonin) receptor 1D	−1.0	0.842
66	Rn.44301	NM_021857	*Htr1f*	5-hydroxytryptamine (serotonin) receptor 1F	1.1	0.848
67	Rn.10294	NM_017254	*Htr2a*	5-hydroxytryptamine (serotonin) receptor 2A	1.2	0.599
68	Rn.9935	NM_012765	*Htr2c*	5-hydroxytryptamine (serotonin) receptor 2C	−1.1	0.706
69	Rn.55109	NM_024394	*Htr3a*	5-hydroxytryptamine (serotonin) receptor 3a	−1.2	0.519
70	Rn.10094	NM_012853	*Htr4*	5-hydroxytryptamine (serotonin) receptor 4	1.0	0.903
71	Rn.87132	NM_022938	*Htr7*	5-hydroxytryptamine (serotonin) receptor 7	−1.1	0.736
72	Rn.64505	NM_023968	*Npy2r*	Neuropeptide Y receptor Y2	−1.0	0.842
73	Rn.10532	NM_012869	*Npy5r*	Neuropeptide Y receptor Y5	−1.0	0.740
74	Rn.127792	NM_022695	*Ntsr2*	Neurotensin receptor 2	−1.1	0.505
75	Rn.6841	NM_012871	*Oxtr*	Oxytocin receptor	1.0	0.874
76	Rn.82760	NM_138978	*Prokr2*	Prokineticin receptor 2	1.0	0.828
77	Rn.32256	NM_031115	*Sctr*	Secretin receptor	−1.3	0.655
78	Rn.42915	NM_012719	*Sstr1*	Somatostatin receptor 1	1.1	0.761
79	Rn.9929	NM_019348	*Sstr2*	Somatostatin receptor 2	1.3	0.084
80	Rn.9936	NM_013036	*Sstr4*	Somatostatin receptor 4	1.2	0.436
81	Rn.89609	NM_012667	*Tacr1*	Tachykinin receptor 1	1.1	0.531
82	Rn.202846	NM_080768	*Tacr2*	Tachykinin receptor 2	−1.4	0.297
83	Rn.9702	NM_017053	*Tacr3*	Tachykinin receptor 3	1.4	0.265
84	Rn.1820	NM_012515	*Tspo*	Translocator protein	−1.1	0.666

**Table 4:** Intermittent HFD exposure and rat neurotransmitter receptors gene expression in the striatum using the RT^2^ Profiler PCR Array. The bold underlined text highlights statistically significant changes compared to the chow controls.

#	Unigene	Refseq	Symbol	Description	Fold Regulation	*p* Value
1	Rn.9991	NM_017191	*Adra1a*	Adrenergic, alpha-1A-, receptor	−1.3	0.037
2	Rn.11314	NM_024483	*Adra1d*	Adrenergic, alpha-1D-, receptor	−5.9	0.037
3	Rn.170171	NM_012739	*Adra2a*	Adrenergic, alpha-2A-, receptor	−1.7	0.038
4	Rn.10206	NM_012492	*Adrb2*	Adrenergic, beta-2-, receptor, surface	1.0	0.842
5	Rn.10100	NM_013108	*Adrb3*	Adrenergic, beta-3-, receptor	−1.1	0.455
6	Rn.32282	NM_053019	*Avpr1a*	Arginine vasopressin receptor 1A	−1.2	0.467
7	Rn.10096	NM_017205	*Avpr1b*	Arginine vasopressin receptor 1B	1.8	0.208
8	Rn.86415	NM_152845	*Brs3*	Bombesin-like receptor 3	1.2	0.615
9	Rn.90997	NM_013165	*Cckbr*	Cholecystokinin B receptor	−1.3	0.321
10	Rn.119395	NM_080773	*Chrm1*	Cholinergic receptor, muscarinic 1	−1.2	0.409
11	Rn.10676	NM_031547	*Chrm4*	Cholinergic receptor, muscarinic 4	−1.1	0.926
12	Rn.44293	NM_017362	*Chrm5*	Cholinergic receptor, muscarinic 5	2.0	0.042
13	Rn.10996	NM_052805	*Chrna3*	Cholinergic receptor, nicotinic, alpha 3	3.1	0.040
14	Rn.9697	NM_024354	*Chrna4*	Cholinergic receptor, nicotinic, alpha 4	−3.2	0.027
15	Rn.40125	NM_017078	*Chrna5*	Cholinergic receptor, nicotinic, alpha 5	−3.5	0.027
16	Rn.9696	NM_057184	*Chrna6*	Cholinergic receptor, nicotinic, alpha 6	−1.5	0.101
17	Rn.9698	NM_012832	*Chrna7*	Cholinergic receptor, nicotinic, alpha 7	−1.2	0.220
18	Rn.10301	NM_017194	*Chrne*	Cholinergic receptor, nicotinic, epsilon	1.7	0.077
19	Rn.89774	NM_012784	*Cnr1*	Cannabinoid receptor 1 (brain)	1.1	0.778
20	Rn.24039	NM_012546	*Drd1*	Dopamine receptor D1A	1.0	0.755
21	Rn.87299	NM_012547	*Drd2*	Dopamine receptor D2	1.1	0.611
22	Rn.138110	NM_012768	*Drd5*	Dopamine receptor D5	−2.0	0.000
23	Rn.30059	NM_031028	*Gabbr1*	Gamma-aminobutyric acid (GABA) B receptor 1	−1.0	0.902
24	Rn.162814	NM_031802	*Gabbr2*	Gamma-aminobutyric acid (GABA) B receptor 2	−2.2	0.002
25	Rn.28463	NM_183326	*Gabra1*	Gamma-aminobutyric acid (GABA) A receptor, alpha 1	−1.1	0.872
26	Rn.48180	NM_001135779	*Gabra2*	Gamma-aminobutyric acid (GABA-A) receptor, subunit alpha 2	1.1	0.340
27	Rn.81205	NM_080587	*Gabra4*	Gamma-aminobutyric acid (GABA) A receptor, alpha 4	1.2	0.155
28	Rn.10368	NM_017295	*Gabra5*	Gamma-aminobutyric acid (GABA) A receptor, alpha 5	−1.7	0.009
29	Rn.29890	NM_021841	*Gabra6*	Gamma-aminobutyric acid (GABA) A receptor, alpha 6	3.3	0.434
30	Rn.207157	NM_012956	*Gabrb1*	Gamma-aminobutyric acid (GABA) A receptor, beta 1	1.1	0.629
31	Rn.233948	NM_017065	*Gabrb3*	Gamma-aminobutyric acid (GABA) A receptor, beta 3	−1.0	0.998
32	Rn.10927	NM_017289	*Gabrd*	Gamma-aminobutyric acid (GABA) A receptor, delta	1.0	0.652
33	Rn.54455	NM_023091	*Gabre*	Gamma-aminobutyric acid (GABA) A receptor, epsilon	1.4	0.593
34	Rn.10366	NM_080586	*Gabrg1*	Gamma-aminobutyric acid (GABA) A receptor, gamma 1	1.2	0.123
35	Rn.230132	NM_183327	*Gabrg2*	Gamma-aminobutyric acid (GABA) A receptor, gamma 2	1.0	0.941
36	Rn.10369	NM_024370	*Gabrg3*	Gamma-aminobutyric acid (GABA) A receptor, gamma 3	1.2	0.377
37	Rn.81067	NM_031733	*Gabrq*	Gamma-aminobutyric acid (GABA) receptor, theta	1.0	0.887
38	Rn.33552	NM_017291	*Gabrr1*	Gamma-aminobutyric acid (GABA) receptor, rho 1	−1.2	0.250
39	Rn.48659	NM_017292	*Gabrr2*	Gamma-aminobutyric acid (GABA) receptor, rho 2	−1.6	0.278
40	Rn.11225	NM_172092	*Gcgr*	Glucagon receptor	1.0	0.838
41	Rn.29971	NM_031608	*Gria1*	Glutamate receptor, ionotropic, AMPA 1	1.0	0.799
42	Rn.91361	NM_017261	*Gria2*	Glutamate receptor, ionotropic, AMPA 2	1.0	0.789
43	Rn.74049	NM_032990	*Gria3*	Glutamate receptor, ionotrophic, AMPA 3	−1.2	0.241
44	Rn.10449	NM_017241	*Grik1*	Glutamate receptor, ionotropic, kainate 1	−1.1	0.394
45	Rn.87696	NM_019309	*Grik2*	Glutamate receptor, ionotropic, kainate 2	1.2	0.054
46	Rn.10049	NM_012572	*Grik4*	Glutamate receptor, ionotropic, kainate 4	−1.1	0.307
47	Rn.74042	NM_031508	*Grik5*	Glutamate receptor, ionotropic, kainate 5	−1.1	0.345
48	Rn.9840	NM_017010	*Grin1*	Glutamate receptor, ionotropic, N-methyl D-aspartate 1	−1.1	0.731
49	Rn.9710	NM_012573	*Grin2a*	Glutamate receptor, ionotropic, N-methyl D-aspartate 2A	−1.2	0.290
50	Rn.9711	NM_012574	*Grin2b*	Glutamate receptor, ionotropic, N-methyl D-aspartate 2B	−1.1	0.769
51	Rn.9709	NM_012575	*Grin2c*	Glutamate receptor, ionotropic, N-methyl D-aspartate 2C	1.4	0.314
52	Rn.87787	NM_017011	*Grm1*	Glutamate receptor, metabotropic 1	1.2	0.317
53	Rn.41715	NM_001105712	*Grm3*	Glutamate receptor, metabotropic 3	1.2	0.252
54	Rn.89046	NM_022666	*Grm4*	Glutamate receptor, metabotropic 4	1.1	0.290
55	Rn.29972	NM_017012	*Grm5*	Glutamate receptor, metabotropic 5	1.2	0.382
56	Rn.44615	NM_022920	*Grm6*	Glutamate receptor, metabotropic 6	1.1	0.796
57	Rn.10409	NM_031040	*Grm7*	Glutamate receptor, metabotropic 7	1.0	0.717
58	Rn.44420	NM_022202	*Grm8*	Glutamate receptor, metabotropic 8	−1.5	0.093
59	Rn.10316	NM_012706	*Grpr*	Gastrin releasing peptide receptor	1.3	0.359
60	Rn.9893	NM_013074	*Hcrtr2*	Hypocretin (orexin) receptor 2	1.1	0.650
61	Rn.81032	NM_017018	*Hrh1*	Histamine receptor H 1	−1.2	0.250
62	Rn.162272	NM_131909	*Hrh4*	Histamine receptor H4	2.3	0.044
63	Rn.44486	NM_012585	*Htr1a*	5-hydroxytryptamine (serotonin) receptor 1A	−1.1	0.674
64	Rn.138109	NM_022225	*Htr1b*	5-hydroxytryptamine (serotonin) receptor 1B	1.3	0.122
65	Rn.34834	NM_012852	*Htr1d*	5-Hydroxytryptamine (serotonin) receptor 1D	1.4	0.102
66	Rn.44301	NM_021857	*Htr1f*	5-hydroxytryptamine (serotonin) receptor 1F	1.2	0.076
67	Rn.10294	NM_017254	*Htr2a*	5-hydroxytryptamine (serotonin) receptor 2A	−1.2	0.702
68	Rn.9935	NM_012765	*Htr2c*	5-hydroxytryptamine (serotonin) receptor 2C	−1.1	0.672
69	Rn.55109	NM_024394	*Htr3a*	5-hydroxytryptamine (serotonin) receptor 3a	−1.0	0.561
70	Rn.10094	NM_012853	*Htr4*	5-hydroxytryptamine (serotonin) receptor 4	1.3	0.196
71	Rn.87132	NM_022938	*Htr7*	5-hydroxytryptamine (serotonin) receptor 7	1.3	0.530
72	Rn.64505	NM_023968	*Npy2r*	Neuropeptide Y receptor Y2	1.3	0.438
73	Rn.10532	NM_012869	*Npy5r*	Neuropeptide Y receptor Y5	−1.1	0.397
74	Rn.127792	NM_022695	*Ntsr2*	Neurotensin receptor 2	1.1	0.073
75	Rn.6841	NM_012871	*Oxtr*	Oxytocin receptor	−1.1	0.563
76	Rn.82760	NM_138978	*Prokr2*	Prokineticin receptor 2	−1.6	0.096
77	Rn.32256	NM_031115	*Sctr*	Secretin receptor	−3.1	0.073
78	Rn.42915	NM_012719	*Sstr1*	Somatostatin receptor 1	−2.4	0.023
79	Rn.9929	NM_019348	*Sstr2*	Somatostatin receptor 2	−2.7	0.002
80	Rn.9936	NM_013036	*Sstr4*	Somatostatin receptor 4	−1.1	0.448
81	Rn.89609	NM_012667	*Tacr1*	Tachykinin receptor 1	1.1	0.727
82	Rn.202846	NM_080768	*Tacr2*	Tachykinin receptor 2	2.6	0.316
83	Rn.9702	NM_017053	*Tacr3*	Tachykinin receptor 3	1.1	0.757
84	Rn.1820	NM_012515	*Tspo*	Translocator protein	1.2	0.336

**Table 5:** Intermittent HFD exposure and rat neurotransmitter receptors gene expression in the ventral tegmental area (VTA) using the RT^2^ Profiler PCR Array. The bold underlined text highlights statistically significant changes compared to the chow controls.

#	Unigene	Refseq	Symbol	Description	Fold Regulation	*p* Value
1	Rn.9991	NM_017191	*Adra1a*	Adrenergic, alpha-1A-, receptor	−1.0	0.955
2	Rn.11314	NM_024483	*Adra1d*	Adrenergic, alpha-1D-, receptor	−1.9	0.436
3	Rn.170171	NM_012739	*Adra2a*	Adrenergic, alpha-2A-, receptor	−1.5	0.170
4	Rn.10206	NM_012492	*Adrb2*	Adrenergic, beta-2-, receptor, surface	−1.2	0.478
5	Rn.32282	NM_053019	*Avpr1a*	Arginine vasopressin receptor 1A	1.0	0.745
6	Rn.10096	NM_017205	*Avpr1b*	Arginine vasopressin receptor 1B	1.2	0.681
7	Rn.86415	NM_152845	*Brs3*	Bombesin-like receptor 3	1.1	0.598
8	Rn.90997	NM_013165	*Cckbr*	Cholecystokinin B receptor	−1.2	0.882
9	Rn.119395	NM_080773	*Chrm1*	Cholinergic receptor, muscarinic 1	1.2	0.858
10	Rn.10676	NM_031547	*Chrm4*	Cholinergic receptor, muscarinic 4	1.4	0.434
11	Rn.44293	NM_017362	*Chrm5*	Cholinergic receptor, muscarinic 5	3.0	0.082
12	Rn.10996	NM_052805	*Chrna3*	Cholinergic receptor, nicotinic, alpha 3	2.1	0.027
13	Rn.9697	NM_024354	*Chrna4*	Cholinergic receptor, nicotinic, alpha 4	−1.2	0.021
14	Rn.40125	NM_017078	*Chrna5*	Cholinergic receptor, nicotinic, alpha 5	10.8	0.037
15	Rn.9696	NM_057184	*Chrna6*	Cholinergic receptor, nicotinic, alpha 6	22.2	0.302
16	Rn.9698	NM_012832	*Chrna7*	Cholinergic receptor, nicotinic, alpha 7	1.2	0.627
17	Rn.10301	NM_017194	*Chrne*	Cholinergic receptor, nicotinic, epsilon	−1.4	0.054
18	Rn.89774	NM_012784	*Cnr1*	Cannabinoid receptor 1 (brain)	−1.6	0.289
19	Rn.24039	NM_012546	*Drd1*	Dopamine receptor D1A	1.6	0.228
20	Rn.87299	NM_012547	*Drd2*	Dopamine receptor D2	3.5	0.248
21	Rn.138110	NM_012768	*Drd5*	Dopamine receptor D5	−1.2	0.410
22	Rn.30059	NM_031028	*Gabbr1*	Gamma-aminobutyric acid (GABA) B receptor 1	−1.0	0.272
23	Rn.162814	NM_031802	*Gabbr2*	Gamma-aminobutyric acid (GABA) B receptor 2	−1.3	0.191
24	Rn.28463	NM_183326	*Gabra1*	Gamma-aminobutyric acid (GABA) A receptor, alpha 1	−1.2	0.097
25	Rn.48180	NM_001135779	*Gabra2*	Gamma-aminobutyric acid (GABA-A) receptor, subunit alpha 2	1.0	0.729
26	Rn.81205	NM_080587	*Gabra4*	Gamma-aminobutyric acid (GABA) A receptor, alpha 4	1.8	0.154
27	Rn.10368	NM_017295	*Gabra5*	Gamma-aminobutyric acid (GABA) A receptor, alpha 5	−1.6	0.080
28	Rn.29890	NM_021841	*Gabra6*	Gamma-aminobutyric acid (GABA) A receptor, alpha 6	1.3	0.499
29	Rn.207157	NM_012956	*Gabrb1*	Gamma-aminobutyric acid (GABA) A receptor, beta 1	1.3	0.031
30	Rn.233948	NM_017065	*Gabrb3*	Gamma-aminobutyric acid (GABA) A receptor, beta 3	−1.1	0.118
31	Rn.10927	NM_017289	*Gabrd*	Gamma-aminobutyric acid (GABA) A receptor, delta	1.2	0.464
32	Rn.54455	NM_023091	*Gabre*	Gamma-aminobutyric acid (GABA) A receptor, epsilon	1.2	0.424
33	Rn.10366	NM_080586	*Gabrg1*	Gamma-aminobutyric acid (GABA) A receptor, gamma 1	−1.1	0.210
34	Rn.230132	NM_183327	*Gabrg2*	Gamma-aminobutyric acid (GABA) A receptor, gamma 2	−1.3	0.158
35	Rn.10369	NM_024370	*Gabrg3*	Gamma-aminobutyric acid (GABA) A receptor, gamma 3	1.6	0.096
36	Rn.81067	NM_031733	*Gabrq*	Gamma-aminobutyric acid (GABA) receptor, theta	−1.1	0.938
37	Rn.33552	NM_017291	*Gabrr1*	Gamma-aminobutyric acid (GABA) receptor, rho 1	2.2	0.044
38	Rn.48659	NM_017292	*Gabrr2*	Gamma-aminobutyric acid (GABA) receptor, rho 2	−1.2	0.978
39	Rn.11225	NM_172092	*Gcgr*	Glucagon receptor	1.4	0.359
40	Rn.29971	NM_031608	*Gria1*	Glutamate receptor, ionotropic, AMPA 1	1.4	0.071
41	Rn.91361	NM_017261	*Gria2*	Glutamate receptor, ionotropic, AMPA 2	1.0	0.786
42	Rn.74049	NM_032990	*Gria3*	Glutamate receptor, ionotrophic, AMPA 3	−1.8	0.025
43	Rn.10449	NM_017241	*Grik1*	Glutamate receptor, ionotropic, kainate 1	−1.3	0.280
44	Rn.87696	NM_019309	*Grik2*	Glutamate receptor, ionotropic, kainate 2	1.1	0.477
45	Rn.10049	NM_012572	*Grik4*	Glutamate receptor, ionotropic, kainate 4	−1.2	0.410
46	Rn.74042	NM_031508	*Grik5*	Glutamate receptor, ionotropic, kainate 5	1.0	0.976
47	Rn.9840	NM_017010	*Grin1*	Glutamate receptor, ionotropic, N-methyl D-aspartate 1	−1.3	0.095
48	Rn.9710	NM_012573	*Grin2a*	Glutamate receptor, ionotropic, N-methyl D-aspartate 2A	−1.1	0.359
49	Rn.9711	NM_012574	*Grin2b*	Glutamate receptor, ionotropic, N-methyl D-aspartate 2B	1.1	0.476
50	Rn.9709	NM_012575	*Grin2c*	Glutamate receptor, ionotropic, N-methyl D-aspartate 2C	−1.1	0.193
51	Rn.87787	NM_017011	*Grm1*	Glutamate receptor, metabotropic 1	1.5	0.426
52	Rn.41715	NM_001105712	*Grm3*	Glutamate receptor, metabotropic 3	−1.3	0.157
53	Rn.89046	NM_022666	*Grm4*	Glutamate receptor, metabotropic 4	−1.1	0.186
54	Rn.29972	NM_017012	*Grm5*	Glutamate receptor, metabotropic 5	−1.1	0.696
55	Rn.44615	NM_022920	*Grm6*	Glutamate receptor, metabotropic 6	1.5	0.240
56	Rn.10409	NM_031040	*Grm7*	Glutamate receptor, metabotropic 7	−1.1	0.569
57	Rn.44420	NM_022202	*Grm8*	Glutamate receptor, metabotropic 8	−1.8	0.231
58	Rn.10316	NM_012706	*Grpr*	Gastrin releasing peptide receptor	1.9	0.326
59	Rn.9893	NM_013074	*Hcrtr2*	Hypocretin (orexin) receptor 2	−1.6	0.151
60	Rn.81032	NM_017018	*Hrh1*	Histamine receptor H 1	−1.8	0.113
61	Rn.162272	NM_131909	*Hrh4*	Histamine receptor H4	1.1	0.616
62	Rn.44486	NM_012585	*Htr1a*	5-hydroxytryptamine (serotonin) receptor 1A	1.7	0.004
63	Rn.138109	NM_022225	*Htr1b*	5-hydroxytryptamine (serotonin) receptor 1B	1.6	0.032
64	Rn.34834	NM_012852	*Htr1d*	5-Hydroxytryptamine (serotonin) receptor 1D	1.3	0.452
65	Rn.44301	NM_021857	*Htr1f*	5-hydroxytryptamine (serotonin) receptor 1F	−1.5	0.073
66	Rn.10294	NM_017254	*Htr2a*	5-hydroxytryptamine (serotonin) receptor 2A	1.0	0.761
67	Rn.9935	NM_012765	*Htr2c*	5-hydroxytryptamine (serotonin) receptor 2C	−1.1	0.561
68	Rn.55109	NM_024394	*Htr3a*	5-hydroxytryptamine (serotonin) receptor 3a	−1.6	0.112
69	Rn.10094	NM_012853	*Htr4*	5-hydroxytryptamine (serotonin) receptor 4	1.8	0.059
70	Rn.87132	NM_022938	*Htr7*	5-hydroxytryptamine (serotonin) receptor 7	−1.2	0.450
71	Rn.64505	NM_023968	*Npy2r*	Neuropeptide Y receptor Y2	−2.3	0.153
72	Rn.10532	NM_012869	*Npy5r*	Neuropeptide Y receptor Y5	1.0	0.963
73	Rn.127792	NM_022695	*Ntsr2*	Neurotensin receptor 2	1.2	0.069
74	Rn.6841	NM_012871	*Oxtr*	Oxytocin receptor	1.4	0.265
75	Rn.82760	NM_138978	*Prokr2*	Prokineticin receptor 2	−1.3	0.083
76	Rn.32256	NM_031115	*Sctr*	Secretin receptor	−1.9	0.080
77	Rn.42915	NM_012719	*Sstr1*	Somatostatin receptor 1	1.2	0.498
78	Rn.9929	NM_019348	*Sstr2*	Somatostatin receptor 2	1.5	0.046
79	Rn.9936	NM_013036	*Sstr4*	Somatostatin receptor 4	1.3	0.342
80	Rn.89609	NM_012667	*Tacr1*	Tachykinin receptor 1	−1.5	0.325
81	Rn.202846	NM_080768	*Tacr2*	Tachykinin receptor 2	2.8	0.003
82	Rn.9702	NM_017053	*Tacr3*	Tachykinin receptor 3	3.2	0.049
83	Rn.1820	NM_012515	*Tspo*	Translocator protein	1.5	0.177
